# Comparison of Delivery-Related, Early and Late Postpartum Severe Maternal Morbidity Among Individuals With Commercial Insurance in the US, 2016 to 2017

**DOI:** 10.1001/jamanetworkopen.2021.37716

**Published:** 2021-12-08

**Authors:** Lindsay K. Admon, Vanessa K. Dalton, Giselle E. Kolenic, Anca Tilea, Stephanie V. Hall, Katy Backes Kozhimannil, Kara Zivin

**Affiliations:** 1 Department of Obstetrics and Gynecology, University of Michigan Medical School, Ann Arbor; 2 Institute for Healthcare Policy and Innovation, University of Michigan, Ann Arbor; 3 Program on Women’s Healthcare Effectiveness Research, Department of Obstetrics and Gynecology, University of Michigan Medical School, Ann Arbor; 4 Department of Learning Health Sciences, University of Michigan Medical School, Ann Arbor; 5 Department of Health Policy and Management, University of Minnesota School of Public Health, Minneapolis; 6 Department of Psychiatry, University of Michigan Medical School, Ann Arbor; 7 Department of Health Policy and Management, University of Michigan School of Public Health, Ann Arbor; 8 VA Ann Arbor Healthcare System, Ann Arbor, Michigan

## Abstract

This cross-sectional study assesses the relative rates and characteristics of early and late postpartum severe maternal morbidity overall and among racial and ethnic groups and individuals with perinatal mood and anxiety disorder.

## Introduction

The rate of late postpartum deaths is substantial in the US compared with other countries.^1^ Similar to maternal mortality occurring during and shortly after pregnancy, a 2019 report indicated that non-Hispanic Black individuals had the highest risk for late postpartum deaths in the US.^2^ Leading causes of late postpartum deaths differ from those occurring earlier in the perinatal period, with a large proportion resulting from underlying perinatal mood and anxiety disorders (PMADs).^3^ Severe maternal morbidity (SMM) is considered proximate to maternal mortality because without identification and treatment, these conditions would lead to maternal death in some cases. Studies often assess SMM at birth and early postpartum to develop strategies to prevent maternal mortality. This study assessed relative rates and characteristics of SMM occurring late postpartum overall, by race and ethnicity, and among those diagnosed with PMADs.

## Methods

This cross-sectional study of deidentified data was considered exempt from review by the University of Michigan Institutional Review Board. The study followed the Strengthening the Reporting of Observational Studies in Epidemiology (STROBE) reporting guideline.

Data were analyzed from December 14, 2020, to October 7, 2021. Using deidentified claims data from the Optum Clinformatics Data Mart (Optum) for 2016 to 2017, we evaluated rates of SMM with and without blood transfusion during 3 pregnancy periods, including hospitalization for birth, hospital discharge to 42 days postpartum, and 43 to 365 days postpartum, among individuals aged 15 to 44 years. We restricted the sample to individuals with continuous enrollment in a single employer-based health plan for at least 1 year before and 1 year after a live birth. For individuals with more than 1 delivery in the 2-year period, we focused only on the index delivery. We identified SMM using diagnosis and procedure codes described by the US Centers for Disease Control and Prevention.^4^ Race and ethnicity were determined based on classifications provided in the Optum database (ie, Asian, Black, Hispanic, White, and unknown/missing), and PMADs were identified in delivery records.^5^

We assessed rates of SMM with and without blood transfusion in each period using logistic regression models with predictive margins adjusted for age, insurance plan type, region, obstetric comorbidity,^6^ race and ethnicity, and PMADs. Cluster-based standard errors accounted for heteroskedasticity across insurance plans. We performed all analyses (2-sided test, α = .05 threshold) using SAS version 9.4 (SAS Institute Inc) and Stata version 14.1 (StataCorp LLC).

## Results

The study sample comprised 100 982 individuals with a mean (SD) age of 31.6 (5.2) years. Rates (95% CIs) of SMM with transfusion were 177.7 (169.6-186.0), 61.1 (56.4-66.1), and 30.3 (27.0-33.9) per 10 000 delivery hospitalizations across the pregnancy, early postpartum, and late postpartum periods, respectively. SMM without blood transfusion followed a similar pattern, with rates (95% CIs) of 105.2 (99.0-111.7), 56.5 (52.0-61.3), and 26.5 (23.5-29.9) per 10 000 delivery hospitalizations, respectively. The frequency of specific SMM indicators varied by period (Table 1).

**Table 1.** Severe Maternal Morbidity Indicator Rates Among 2536 Individuals by Pregnancy Period per 10 000 Delivery Hospitalizations

Characteristic	Birth (n = 1794)		Postpartum			
			Discharge to 42 d (n = 617)		43 to 365 d (n = 306)	
	Indicator	Unadjusted rate (95% CI)	Indicator	Unadjusted rate (95% CI)	Indicator	Unadjusted rate (95% CI)
SMM composite outcome						
With blood transfusion	NA	177.7 (169.6-186.0)	NA	61.1 (56.4-66.1)	NA	30.3 (27.0-33.9)
Without blood transfusion	NA	105.2 (99.0-111.7)	NA	56.5 (52.0-61.3)	NA	26.5 (23.5-29.9)
Indicator rank						
1	Blood transfusion	72.5 (67.4-77.9)	Severe anesthesia complications	18.8 (16.2-21.7)	Severe anesthesia complications	6.9 (5.4-8.8)
2	Hysterectomy	57.2 (62.1)	Heart failure/arrest during surgery or procedure	14.7 (12.4-17.2)	Acute kidney failure	5.0 (3.7-6.5)
3	Eclampsia	20.9 (18.2-23.9)	Sepsis	11.6 (9.6-13.9)	Sepsis	4.1 (2.9-5.5)
4	Heart failure/arrest during surgery or procedure	14.9 (12.6-17.4)	Acute kidney failure	5.4 (4.0-7.0)	Blood transfusion	3.8 (2.7-5.2)
5	Acute kidney failure	10.0 (8.2-12.2)	Adult respiratory distress syndrome	5.2 (3.9-6.8)	Adult respiratory distress syndrome	3.6 (2.5-4.9)

Abbreviations: NA, not applicable; SMM, severe maternal morbidity.

Rates of SMM with and without blood transfusion varied by race and ethnicity and PMAD status. Higher rates (95% CIs) of SMM with transfusion were identified among Black individuals (277.3 [245.2-309.4], 110.7 [88.3-133.1], and 44.3 [30.0-58.6]) compared with White individuals (166.7 [157.0-176.4], 56.4 [50.5-62.3], and 28.9 [24.7-33.2]) for the pregnancy, early postpartum, and late postpartum periods, respectively. Higher rates (95% CIs) of SMM with transfusion were also identified in each of the 3 periods among individuals with PMADs (339.2 [294.8-383.7], 167.7 [136.3-199.2], and 131.8 [103.2-160.5]) compared with individuals without PMADs (166.2 [157.9-174.5], 53.7 [49.1-58.3], and 23.5 [20.3-26.7]), respectively (Table 2).

**Table 2.** Severe Maternal Morbidity by Race and Ethnicity and Mental Health Comorbidity Among 100 982 Commercially Insured Individuals

Characteristic	Adjusted rate (95% CI)						
	Race and ethnicity^a^					Presence of PMAD^b^	
	White [reference]	Asian	Black	Hispanic	Unknown/missing	No [reference]	Yes
No. of participants	64 744	7103	8088	12 850	8197	95 321	5661
SMM with blood transfusion							
Hospitalization for birth	166.7 (157.0-176.4)	144.9 (117.2-172.5)	277.3 (245.2-309.4)^c^	187.1 (161.8-212.5)	155.9 (127.4-184.4)	166.2 (157.9-174.5)	339.2 (294.8-383.7)^c^
Postpartum							
Discharge to 42 d	56.4 (50.5-62.3)	57.5 (40.7-74.3)	110.7 (88.3-133.1)^c^	60.3 (47.6-73.1)	45.4 (30.7-60.2)	53.7 (49.1-58.3)	167.7 (136.3-199.2)^c^
43 to 365 d	28.9 (24.7-33.2)	20.7 (8.0-33.4)	44.3 (30.0-58.6)^d^	38.2 (26.7-49.8)	21.1 (11.3-30.8)	23.5 (20.3-26.7)	131.8 (103.2-160.5)^c^
SMM without blood transfusion							
Hospitalization for birth	102.2 (94.3-100.0)	65.7 (46.8-84.6)^e^	173.1 (148.7-197.4)^c^	106.0 (87.3-124.7)	74.8 (55.2-94.4)^d^	96.2 (90.0-102.4)	222.8 (187.6-258.0)^c^
Postpartum							
Discharge to 42 d	51.5 (45.9-57.2)	52.5 (35.4-69.6)	105.3 (83.6-127.0)^c^	56.7 (44.5-69.0)	42.9 (28.6-57.2)	50.1 (45.8-54.5)	148.1 (118.8-177.4)^c^
43 to 365 d	25.7 (21.7-29.7)	18.7 (6.7-30.8)	40.6 (27.2-54.0)^d^	32.9 (21.8-43.9)	14.2 (6.2-22.2)^d^	20.7 (17.8-23.7)	112.8 (86.2-139.4)^c^

Abbreviations: PMAD, perinatal mood and anxiety disorder; SMM, severe maternal morbidity.

^a^ Model estimating rates by race and ethnicity adjusted for age, insurance plan type, income, region, PMAD diagnosis, and obstetric comorbidity.

^b^ Model estimating rates by PMAD comorbidity adjusted for age, race and ethnicity, income, insurance plan type region, and obstetric comorbidity.

^c^ *P* < .001.

^d^ *P* < .05.

^e^ *P* < .01.

## Discussion

The most common SMM indicators early and late postpartum included severe anesthesia complications and sepsis. Black individuals had the highest rates of SMM in each pregnancy period compared to individuals from other racial and ethnic groups. In contrast with trends in the overall population, individuals with PMADs experienced a markedly sustained risk for SMM early and late postpartum, which should inform clinical and policy strategies to address and prevent maternal morbidity and mortality among this population.

Limitations of this study include the following: the focus on live births rather than other pregnancy outcomes; the limited generalizability of a study population with continuous commercial insurance coverage during the postpartum year; the inability to measure death as a competing outcome, given the low sample size; issues inherent with using the race and ethnicity categories provided in the Optum database; and the inability to measure racism. Nonetheless, our findings suggest that racial and ethnic inequities are associated with SMM in the postpartum year and that SMM is particularly elevated among individuals with PMADs.
